# A case of Bowen’s disease possibly attributed to chronic stimulation by a metal wristwatch^☆^

**DOI:** 10.1016/j.abd.2021.02.016

**Published:** 2022-11-24

**Authors:** Maki Takada, Masato Ishikawa, Yuka Hanami, Toshiyuki Yamamoto

**Affiliations:** Department of Dermatology, Fukushima Medical University, Fukushima, Japan

Dear Editor,

Bowen’s disease, Squamous Cell Carcinoma (SCC) in situ, is a common skin cancer in elderly people, and its causal factors include sun exposure, irradiation, arsenic exposure, burn, scar, injury, Human Papillomavirus (HPV), and immunosuppressive status. However, Bowen’s disease occurring on chronically stimulated sites has rarely been reported.^1^ We report herein a case of Bowen’s disease on the wrist possibly caused by chronic stimulation by contact with the patient’s metal wristwatch.

An 82-year-old man was referred to our hospital after presenting with erythema and erosion on his left wrist where he wore his wristwatch for over fifty years. He had had the lesion for 10 years, and it had gradually increased in size. Physical examination revealed a single 35 × 23 mm lesion that appeared as a well-defined erythematous plaque on the dorsum of his left wrist, which had erosion and crust (Fig. 1). The lesion was about the same size as the area covered by his wristwatch. A biopsy was performed and the histological examination revealed irregular acanthosis, full-thickness dysplasia of the epidermis, pleomorphism with hyperchromatic nuclei, and numerous mitoses. There was no invasion of atypical cells into the dermis. These histological features were consistent with those of squamous cell carcinoma in situ or Bowen’s disease (Fig. 2A‒B). As the patient did not want surgery, he was started on topical imiquimod 5% cream daily for 3 months, and the erythema and erosion of the lesion were reduced. 5-Fluorouracil cream was thereafter administered for 13 months, and the patient stopped using it of his own volition due to the complete improvement of the lesion.

**Figure 1:**
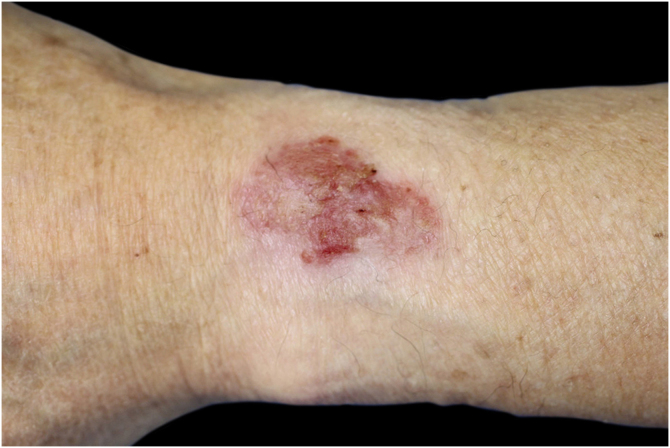
Clinical features showing well-defined erythematous plaque with erosion and crusts in the area covered by the metal wristwatch on the left wrist.

**Figure 2:**
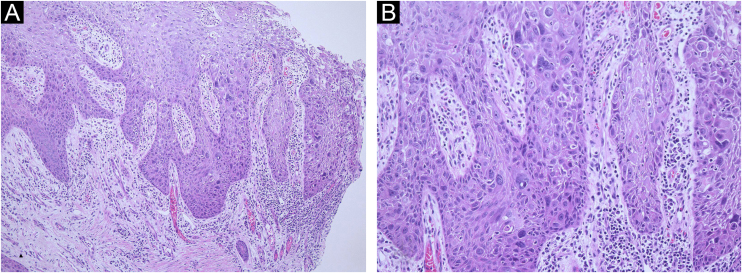
(A) Low power view of a biopsy specimen from the wrist shows irregular acanthosis and full-thickness dysplasia of the epidermis without infiltration of atypical cells into the dermis. (Hematoxylin & eosin, ×100). (B) Higher magnification shows the epidermis with hyperchromatic nuclei, atypia, and numerous mitoses. (Hematoxylin & eosin, ×200).

The patient had Bowen’s disease that may have been caused by chronic stimulation as a result of repetitive contact or friction with the case back of his metal wristwatch. The location of the lesion corresponded to the site as well as the size in contact with the metal on a daily basis. Although a metal patch test was not performed, we believe that the occurrence of Bowen’s disease was related not to a metal allergy, but to long-term chronic stimulation.

Chronic stimulation leads to chronic wounds and scars, which can develop into squamous cell carcinoma.^2^ It has been reported that when a scar is formed, it interferes with the immune response, which leads to the formation of a tumor.^3^ This mechanism may be associated with the development of Bowen’s disease. Previously, a case of Bowen’s disease arising on an old scar site was reported, in which Bowen’s disease occurred at the site of a scar from an injury caused by a tin can as long as 50 years previously.^1^

It was reported that cutaneous scars subsequent to burns, radiation, trauma, and vaccination, are vulnerable sites for the development of neoplasms.^4^ Chronic stimulation or dental metal allergy have been reported as causes of oral SCC. In a report by Weber et al., among the 65 patients with oral SCC, 34% showed an allergic reaction to at least one metal that was immediately adjacent to the cancer site. The rate was 1.57 times higher than that of the controls.^5^

In the present case, we speculate that superficial damage may have been induced by chronic stimulation/friction with a metal wristwatch, leading to the development of Bowen’s disease during the repetitive repair process. The current case suggests that even chronic minor stimulation or friction without penetrating trauma or dermal injury may cause Bowen’s disease if repeated for a long time. Further studies are needed to clarify the causative mechanisms of Bowen’s disease.

## Financial support

None declared.

## Authors' contributions

Maki Takada wrote the initial draft of the manuscript. Toshiyuki Yamamoto assisted in the preparation of the manuscript. Masato Ishikawa and Yuka Hanami performed data collection, analysis, and interpretation. All authors have read and approved the final version of the manuscript.

## Conflicts of interest

None declared.
